# Do human B-lymphocytes avoid aging until 60 years?

**DOI:** 10.18632/oncotarget.10146

**Published:** 2016-06-17

**Authors:** Andrea Knight, Pavel Nemec, Sona Bretzova, Lucie Valkova, Marketa Kolmanova, Renata Vytopilova, Marek Havelka, Pavla Vsianska, Lucie Rihova, Marta Krejci, Martin Piskacek

**Affiliations:** ^1^ Department of Pathological Physiology, Faculty of Medicine, Masaryk University Brno, Brno, Czech Republic; ^2^ Gamma-Delta T Cell Laboratory, University Hospital Brno, Brno, Czech Republic; ^3^ Department of Clinical Haematology, University Hospital Brno, Brno, Czech Republic; ^4^ Department of Internal Medicine, Haematology and Oncology, University Hospital Brno, Brno, Czech Republic; ^5^ Laboratory of Cancer Biology and Genetics, University Hospital Brno, Brno, Czech Republic

**Keywords:** aging, B cell, naive B cells, IL7R, GEP, Gerotarget

## Abstract

Broad changes in human innate and adaptive immunity are associated with advanced age. The age-related alteration of gene expression was reported for both T and B lymphocytes. We analysed the genome-wide expression profiles (*n*=20) of naive and whole B cell populations from young and early aged healthy donors under 60 years. We revealed large homogeneity of all analysed genome-wide expression profiles but did not identified any significant gene deregulation between young (30-45 years) and early aged healthy donors (50-60 years). We argue that B cells avoid the aging program on molecular level until 60 years of age. Our results demonstrate the potential of hematopoietic stem cells to generate uncompromised lymphocytes in early elderly. These are very encouraging findings for the general health and the immunity maintenance would not need any intervention to naive B cells. Rather, a suitable immune stimulation in healthy body environment warrants further research into aging of older elderly.

## INTRODUCTION

The immunosenescence is a natural and inevitable process characterized by a declined immunity. Lymphocytes play a central role in the establishment and maintenance of protective immunity. The lymphoid progenitor counts decline with the advancing age and their repertoire is less diverse in elderly [1–4]. The aging of B cells on molecular level and increased vulnerability to age-associated malignancies was reported for the advanced elderly [5–11]. Similarly, the B cell aging was observed in mouse model, where it was linked with Interleukin 7 pathway [12–16].

The prevalence of B cell malignancies, such as Chronic Lymphocytic Leukaemia (CLL), Multiple Myeloma (MM) and Diffuse Large B Cell Lymphoma (DLBCL), rapidly increases with advancing age. According to SEER Cancer Statistics, only about 4% of newly diagnosed patients are diagnosed with these malignancies under the age of 44 years (http://seer.cancer.gov/statfacts/html/mulmy.html). The increasing incidence of 12% was reported for the next decade (between 45 and 54 years) and remains constant in a range of 21% to 27% in newly diagnosed patients per decade between 55-85 years of age. The sigmoid course of this incidence has prompted our interest to investigate B cells in the context of aging below the 60 years.

In this study we determined the genome-wide gene deregulation in B cells in early elderly to address the programmed gene deregulation linked with decline of immunity and precondition for aged associated B cell malignancies.

## RESULTS

### Aging in naive B cells (GEP, *n* = 12)

We have collected peripheral blood samples of young (30-45 years) and old (55-60 years) healthy donors. Naive B cells defined as CD19^+^ CD27^−^ were isolated by MACS sorting. The age, gender and purity of each cell sample are shown (Table 1).

**Table 1:** Healthy donors' information and cell sample purity after MACS cell sorting

GEP-N1	Donor	Age	Gender	purity %
	4461-1973	41	male	98,5
	4945-1976	38	male	93,0
	4453-1958	56	male	89,8
	4948-1956	58	male	87,7
				
GEP-N2	Donor	Age	Gender	purity %
	8947-1980	34	male	99,7
	9021-1973	41	female	96,5
	8960-1957	57	male	99,5
	9025-1954	60	female	99,0
				
GEP-N3	Donor	Age	Gender	purity %
	3556-1984	31	male	99,1
	3885-1985	30	male	99,4
	3521-1957	58	male	99,5
	4402-1957	58	female	99,2
				
GEP-B1	Donor	Age	Gender	purity %
	8947-1980	34	male	92,0
	9021-1973	41	female	96,4
	8960-1957	57	male	95,8
	9025-1954	60	female	95,0
				
GEP-B2	Donor	Age	Gender	purity %
	3556-1984	31	male	99,1
	3885-1985	30	male	99,0
	3521-1957	58	male	99,6
	4402-1957	58	female	99,5

**Figure 1:**
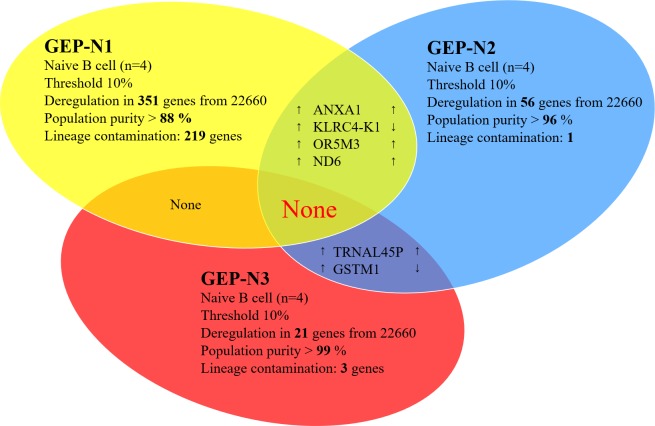
Aging in naive cells (genome-wide profiles, *n* = 12) Venn diagram of results from whole-genome experimental sets GEP-N1, GEP-N2 and GEP-N3 for age-specific genes in naive B cells. The three independent experiments have been conducted, each comprised of four samples have been analysed. Arrows indicate expression upregulation and downregulation with age.

The age-specific genome-wide analyses (*n* = 12) have been determined in three independent sets named GEP-N1, GEP-N2 and GEP-N3, each consisting of four samples (Figure 1). The data for age-specific gene deregulation were collected with the threshold of 10% fold change between young and old donors. From a total of 22,660 annotated genes, we detected 351 deregulated genes for GEP-N1 set, 56 genes for GEP-N2, and 21 genes for GEP-N3. All non-coding RNA, pseudogenes and olfactory receptors were excluded from our analyses.

The samples in GEP-N1 set had the lowest purity (88 %) and therefore were positive also for genes that are expressed in other cell populations including monocytes, T or NK cells. According to the BioGPS database (http://biogps.org/#goto = genereport&id = 22914), genes such as KLRK1 and Annexin A1 are expressed about 160 fold more in CD8 (T cells) and CD33 (myeloid cells) than in B cells. The genes of lineage-specific contamination in our samples were not detected in sets with higher purity (GEP-N2 set with 96% purity and GEP-N3 set with 99% purity). The summary result of genome-wide profiles in the GEP-N1, GEP-N2 and GEP-N3 sets involving naive B cell did not revealed any shared gene (Figure 1).

### Gender specific gene in naive B cells (GEP, *n* = 3 female + 5 male)

**Figure 2:**
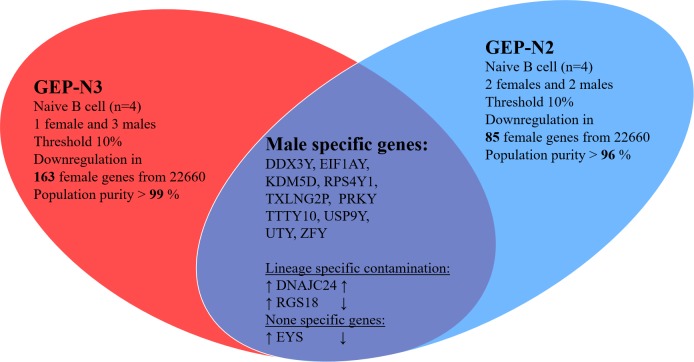
Gender specific gene in naive cells (genome-wide profiles, *n* = 8) Venn diagram of results from whole-genome experimental sets GEP-N2 and GEP-N3 for gender specific genes in naive B cells. In two independent sets, three females and five male samples were investigated. Arrows indicate expression upregulation and downregulation in male.

Next, we aimed to detect gender specific genes in naïve B cells to justify credibility of our samples. The DDX3Y, EIF1AY, KDM5D and RPS4Y1 were reported as gender specific genes in B lymphocytes [17] [18]. We analysed the expression of gender specific genes in male (*n* = 5) *versus* female (*n* = 3) in sets GEP-N2 and GEP-N3 (Figure 2). Of note, GEP-N1 set consisted only of male samples and therefore has not been used here.

We identified several genes including DDX3Y, EIF1AY, KDM5D, RPS4Y1, PRKY, TTY10, TXLNG2P, USP9Y, UTY, and ZFY that are expressed from narrow chromosomal bands Yq11.2 and Yq11.221-223 located on chromosome Y, two genes DNAJC24, RGS18 as lineage-specific contamination according to the expression data in BioGPS database and gene EYS located on chromosome 6 as a representing noise in our experiment.

### Male specific aging in naive B cells (GEP, *n* = 9)

Hypothetically, aging could differ in males and females. Therefore we excluded all females from sets GEP-N1, GEP-N2 and GEP-N3.

**Figure 3:**
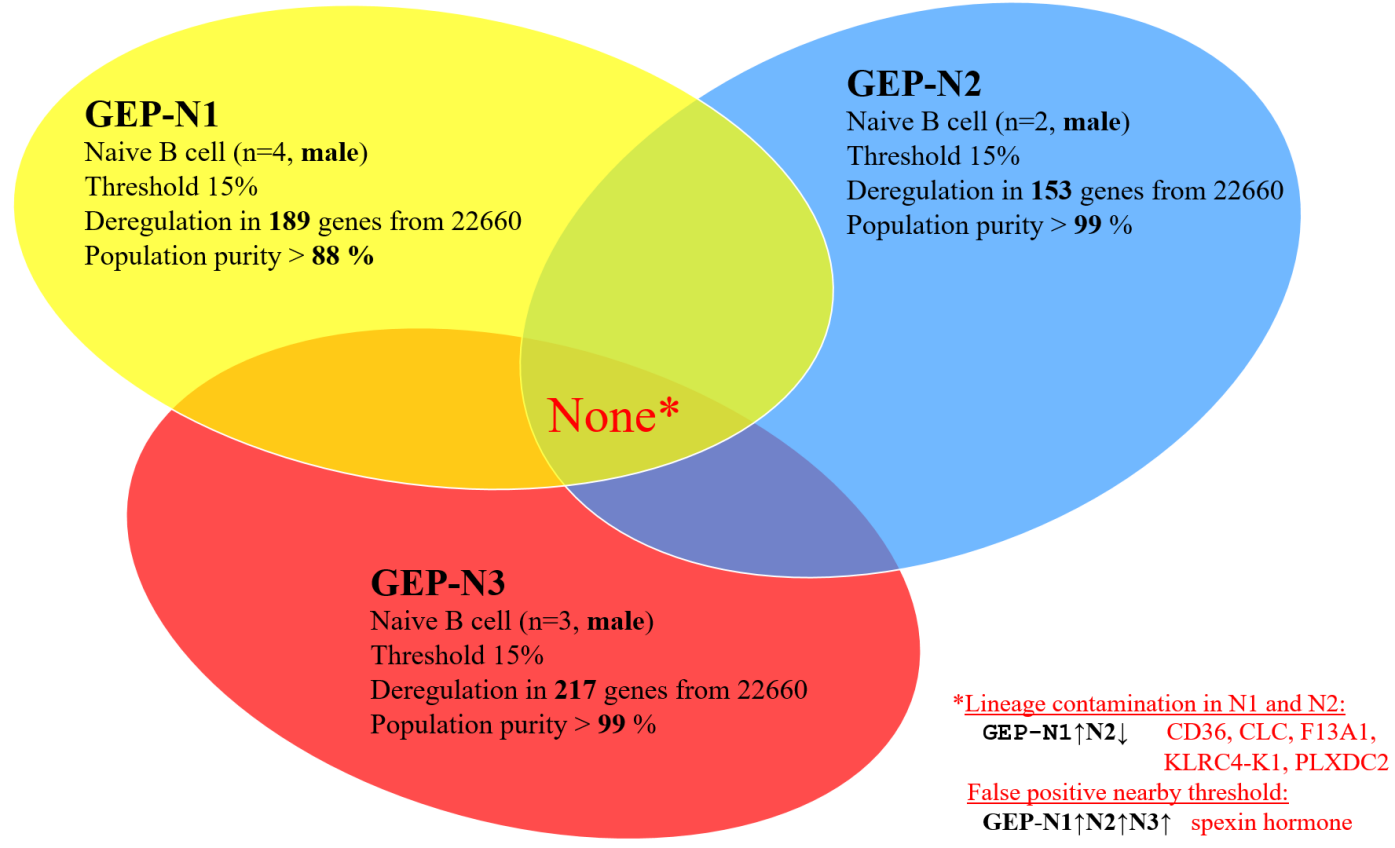
Male specific aging in naive B cells (genome-wide profiles, *n* = 9) Venn diagram of results from whole-genome experimental sets GEP-N1, GEP-N2 and GEP-N3 for male specific aging in naive B cells. In the three independent sets, nine male samples were investigated. Arrows indicate expression upregulation and downregulation with age.

In the gender specific experimental set, male samples (*n* = 9) were included and 15% fold change threshold was applied. The resulting six deregulated genes were recognized as lineage-specific contamination or as an experimental noise. The CD36, CLC, F13A1, KLRC4-K1, PLXDC2 genes are largely expressed in diverse subpopulations of leukocytes including CD3 (T cells), CD14 (monocytes), CD33 (myeloid cells), CD56 (NK cells) positive cells, eosinophils and basophils, but hardly in B cells (BioGPS). The single gene not linked with lineage-specific contamination was C12orf39 gene coding for neuropeptide Spexin. We evaluated this paracrine hormone as a false positive resulting from noise in our experiments (see bellow for further supporting exclusion evidence in results for B cell population). The analyses of the gender restricted genome-wide profiles in the GEP-N1, GEP-N2 and GEP-N3 sets but did not revealed any shared age-specific gene (Figure 3).

### Aging in B cell population (GEP, *n* = 8)

**Figure 4:**
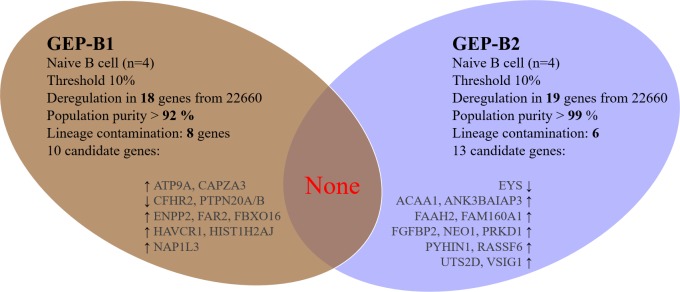
Aging in B cell population (genome-wide profiles, *n* = 8) Venn diagram of results from whole-genome experiments GEP-B2 and GEP-B3 for age-specific genes in B cells. Each independent experiment comprised of four samples, together eight samples were investigated. Arrows indicate expression upregulation and downregulation with age. Arrows indicate expression upregulation and downregulation with age.

We analysed gene deregulation of whole B cell population (*n* = 8) in two independent experimental sets, GEP-B1 and GEP-B2, each consisting of four samples. The analyses of the genome-wide analyses in the GEP-B1 and GEP-B2 sets did not revealed any shared age-specific gene (Figure 4).

### Interleukin 7 receptor signalling in elderly (GEP, *n* = 20; western blotting, *n* = 8)

It has been know that the Interleukin 7 (IL7) signalling declines in elderly as reported in old mice [12, 13]. The IL7 signalling associates with human natural ageing and longevity [8].

**Figure 5:**
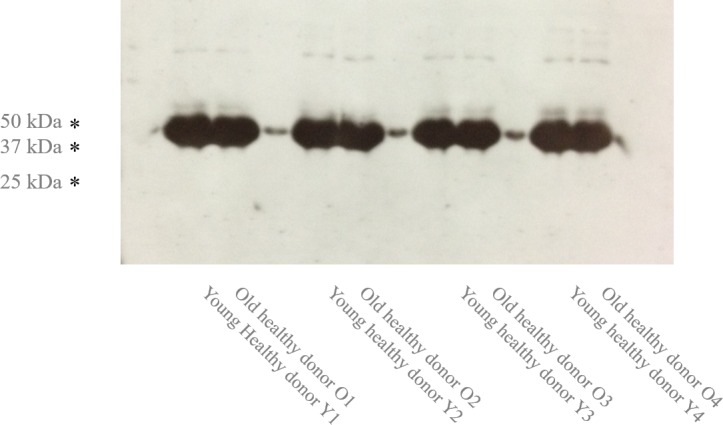
The Age-specific IL7R expression (*n* = 8) Western blotting of four pairs of young and early aged sorted naive B cell samples is shown.

From the animal model, the IL7 responsiveness is linked with the decline of B cell progenitors. Interestingly, gender specific regulation of the IL7 receptor (IL7R) was reported in human B lymphocytes [17]. However, we did not observe any changes in expression of neither targets of IL7R pathway nor IL7R (GEP-N1, N2, N3, B2 and B3). Consistently, we did not observe any IL7R expression changes on protein level (Figure 5).

## DISCUSSION

In this study, we examined age-specific gene deregulated in naive and whole B cell populations in two cohorts of young and early aged healthy donors. We did not identify a single gene to be age-specifically deregulated in our groups of young (30-45 years) and early aged (55-60 years) healthy donors. We observed largely homogenous and overall consistent genome-wide expression in all 20 samples examined. These results strongly indicate that B cells avoid aging program until 60 years of age.

We have excluded non-coding RNAs, olfactory receptors and pseudogenes, although these genes maybe involve in aging process too. The regulatory miRNAs were reported to be age-specifically deregulated in donors exceeding 60 years [5, 19, 20]. In this respect, we conclude from our results, that they did not significantly alter genome-wide expression of annotated protein-coding genes monitored in our analyses (GEP-N1, N2, N3, B2 and B3).

We reviewed the age-specific genes reported by others to be elevated in naive B cell in much older groups of healthy donors with the age over 78 to 90 years including CCR6, CCR7, CXCR4, CXCR5, CD62L, and Granzyme B genes [6].

We identified all these genes in our experimental sets GEP-N1 and GEP-N2 as lineage-specific contamination. Thus we proved age-specific expression of these genes in our GEP-N3 set with the highest overall cell lineage purity to minimize false positivity. The deregulation of age-specific genes listed above is below 4% fold change in the samples from GEP-N3 set and therefore, we considered them as unaltered under 60 years of age.

Significantly, we did not observed any deregulation of B cell lineage promoting factors such as IL7R, E2A, EBF or PAX5 that are associated with B cell decline in humans or animals [12, 13, 21–25]. In general, the age-specific gene deregulation might be longevity-associated and expressed in much older groups of healthy donors with age over 80 years and in centenaries.

As the most of methods, also genome-wide analyses have detection limits, which in turn could limit identification of age-specific genes. Only minor gene alteration might be hidden below the detection threshold and unrecognized in this study. Nevertheless, our genome-wide analysis homogeneity indicated result robustness and confident reliability.

We summarize surprising observations that human B lymphocytes remain almost identical on molecular level during 30 to 60 years of age. As the immune system declines, the predispositions to B cell lineage malignancy manifested in some individuals below 60 years of age could not be addressed to natural healthy aging of B lymphocytes. Rather, other aspects might be involved including compromised body environment, declined cell stimulation, immune cell population disorders, clonal accumulations, infection history, life style and other individual behaviour contributing to early onset of aging [26–28].

The molecular identity of young and early aged B cells demonstrated potential of hematopoietic stem cells to generate uncompromised progenitor lymphocytes, naive and mature B cells in early elderly. These are very encouraging findings for general health, because the immunity maintenance does not seems to needed artificial intervention to keep B cells uncompromised in the early elderly.

As reported previously [26–28], more suitable immune stimulations in healthy body environment was proven advantageous. Further research into aging of older elderly is warranted.

**Figure 6:**
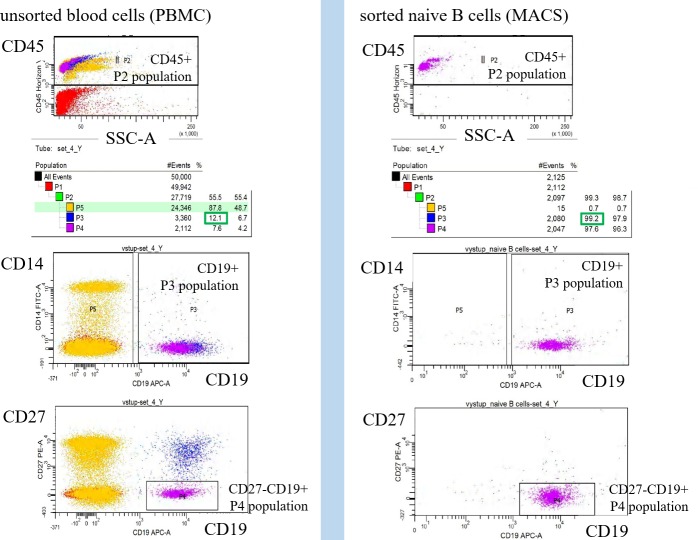
Monitoring of cell population homogeneity by flow cytometry The lineage contamination was monitored by flow cytometry using CD14, CD3, CD45, CD19 and CD27 antibody. The naive B cells homogeneity was defined by ratio of CD19^+^CD27^−^ gated population P4 divided by CD45^+^ gated population P2 (B cells homogeneity = CD19^+^CD27^−^/CD45^+^ = P4/P4).

## MATERIALS AND METHODS

### Cell sorting

Peripheral blood samples were used to isolate the PBMCs by the density gradient centrifugation on Ficoll-Paque according to manufacturer's recommendation (StemCell Tech). Naive B cells were isolated by MACS negative selection with depletion of the CD2, CD14, CD16, CD27, CD36, CD43, CD235a positive cells according to manufacturer's protocol (Miltenyi). B-cell populations were sorted to the highest purity by using MACS positive selection of CD19 cells. Purity of sorted cells was monitored by ration of CD19^+^CD27^−^ to CD45^+^ by flow cytometry.

### Western blotting

The isolated B cells were immediately lysed with loading buffer, incubated 1 min at 95°C, centrifuged 2 min at 14.000g, the extract was loaded on 10% SDS-PAGE, blotted onto nitrocellulose membrane, incubated with 5% low fat milk, and the primary IL7R antibody (St John's Laboratory) and anti-rabbit secondary antibody peroxidase conjugate (Sigma A6667).

### Gene expression analysis (GEP)

Total RNA was isolated from 500 000 cells by QIAGEN RNeasy Mini Kit according to a manufacturer's protocol. RNA concentration was measured by NanoDrop Spectrophotometer, and RNA integrity number (RIN) was calculated using the Agilent 2100 Bioanalyzer (RNA 6000 Nano Kit). Total RNA with absorbance ratio A260/A280 higher than 1.7 and with RIN higher than 7.5 is acceptable for further processing by Ambion WT Expression Kit. Total RNA was used as a template for synthesis of cDNA by reverse transcription. RNA template is degraded using RNase H leaving intact cDNA. Total of 5.5 μg of cDNA is fragmented and labelled using The Affymetrix GeneChip^®^ WT Terminal Labeling Kit. The Human Gene 1.0 ST Array is then washed and stained by Affymetrix fluidics station and scanned by Affymetrix Scanner. Agilent GeneSpring GX 11.5 software for expression data analysis was used. The intensity values (CEL files) were summarised using gene level ExonRMA16 algorithm with quantile normalization and baseline transformation to median of all samples. *T*-test with Benjamini-Hochberg multiple testing correction was used.

Any other details or raw data files are available on the request.
